# Tissue, urine and serum NMR metabolomics dataset from a 5/6 nephrectomy rat model of chronic kidney disease

**DOI:** 10.1016/j.dib.2020.106567

**Published:** 2020-11-23

**Authors:** Munsoor A Hanifa, Martin Skott, Raluca G Maltesen, Bodil S Rasmussen, Søren Nielsen, Jørgen Frøkiær, Troels Ring, Reinhard Wimmer

**Affiliations:** a Department of Chemistry and Bioscience, Aalborg University, 9220 Aalborg, Denmark; b Department of Anaesthesia and Intensive Care Medicine, Aalborg University Hospital, 9000 Aalborg, Denmark; c Department of Clinical Medicine, Aalborg University, 9000 Aalborg, Denmark; d Department of Urology, Aarhus University Hospital, 8250 Aarhus N, Denmark; e 2A Pharma AB, Södergatan 3, 211 34 Malmö, Sweden; f Department of Clinical Medicine, Aarhus University, 8200 Aarhus N, Denmark; g Department of Biomedicine, Aarhus University, 8000 Aarhus C, Denmark; h The Center for Critical Care Nephrology, Department of Critical Care Medicine, University of Pittsburgh, Pittsburg, PA 15261, United States of America

**Keywords:** Chronic kidney disease, NMR, Metabolomics, Rat, 5/6 Nephrectomy

## Abstract

Serum, urine and tissue from a rat model of chronic kidney disease (CKD) were analysed using nuclear magnetic resonance (NMR) spectroscopy-based metabolomics methods, and compared with samples from sham operated rats. Both urine and serum were sampled at multiple timepoints, and the results have been reported elsewhere (https://doi.org/10.1007/s11306-019-1569-3[1]). The data could be useful to researchers working with human CKD or rat models of the disease. In addition, several different types of NMR spectra were recorded, including 1D NOESY, CPMG, and 2D J-resolved spectra, and the data could be useful for method comparison and algorithm development, both in terms of NMR spectroscopy and multivariate analysis.

## Specifications Table

**Table:** 

Subject	Biochemistry
Specific subject area	NMR-based metabolomics in an animal model of chronic kidney disease
Type of data	^1^H NMR spectra
How data were acquired	Bruker 600 MHz Avance DRX-600 NMR spectrometer equipped with a TXI probe
Data format	Raw and processed ^1^H NMR spectra (in Bruker format)
Parameters for data collection	The 5/6 nephrectomy rat model of chronic kidney disease (CKD) was compared to sham operation, by analysing NMR spectra of urine, serum and tissue samples.
Description of data collection	Urine and serum were collected at multiple timepoints after 5/6 nephrectomy or sham operation. Tissue samples (kidney, lung, heart, spleen and liver) were collected when animals were euthanised. Urine and serum were mixed with phosphate buffer, and tissue samples were extracted and reconstituted in phosphate buffer, before ^1^H NMR spectra were acquired.
Data source location	Aalborg University, Aalborg, Denmark
Data accessibility	MetaboLights database (www.ebi.ac.uk/metabolights) accession number MTBLS2052 (www.ebi.ac.uk/metabolights/MTBLS2052)
Related research article	M.A. Hanifa, M. Skott, R.G. Maltesen, B.S. Rasmussen, S. Nielsen, J. Frøkiær, T. Ring, R. Wimmer. Tissue, urine and blood metabolite signatures of chronic kidney disease in the 5/6 nephrectomy rat model. Metabolomics, 15 (2019) 112. https://doi.org/10.1007/s11306-019-1569-3.

## Value of the Data

• The dataset provides a unique combination of tissue, urine and serum NMR spectra from the rat CKD model, as well as a control group for comparison.
• Researchers who are interested in CKD, as well as those interested in metabolomics in general, can benefit from this dataset.
• This dataset can be used as a validation group for other rat CKD studies, or as part of a feasibility study for new rat experiments. The development of the urine metabolomic profile over time could be of interest in planning human experiments.
• Comparison of tissue, urine and serum spectra, and comparison of different types of NMR spectra, may be interesting.

## 1 Data Description

The data consists of raw and processed proton nuclear magnetic resonance (^1^H NMR) spectra, which are in standard Bruker format. NMR spectra were acquired on samples collected from rats after 5/6 nephrectomy or sham operation [1]. There were a total of 147 urine samples, 53 serum samples, and 130 tissue samples, and for each collected sample multiple NMR spectra were recorded using different pulse sequences: 1D nuclear Overhauser effect spectroscopy (NOESY), Carr-Purcell-Meiboom-Gill (CPMG), 2D J-resolved (JRES) including a skyline projection (pJRES), and a quantitative NOESY spectrum using a long relaxation delay (qNOESY). For urine samples all four spectra were recorded; no CPMG spectra were recorded for tissue extracts; and only CPMG and JRES spectra were recorded for serum samples.

Each file has an eight-digit label, read as four pairs of numbers. The first pair represent tissue type, the second pair represent rat number, the third pair represent week number, whilst the fourth pair represent spectral type. Details of the scheme are presented in Table 1. Rats 1, 6, 16, 19, 22, and 23 died before the end of the experiment, mostly during or immediately after the surgical procedures, and therefore no data is available for these rats.

**Table 1:** Naming convention of NMR spectra.

Label digits	Represents	Possible values
First and second	Tissue type	Urine (10), serum (21), remnant kidney (50), left sham kidney (51), right sham kidney (52), lung (60), heart (61), spleen (62), liver (63)
Third and fourth	Rat number	01–30 (no data for rats 01, 06, 16, 19, 22 or 23)
Fifth and sixth	Week number	00–06 (illustrated in Fig. 1)
Seventh and eighth	Spectral type	NOESY (01), CPMG (02), JRES (03), qNOESY (04)

## 2 Experimental Design, Materials and Methods

### 2.1 Animal models and sample collection

Following approval from the Danish Ministry of Justice, 30 male Wistar rats (Taconic, Ejby, Denmark) were randomized, 17 to the 5/6 nephrectomy group and 13 to the sham operation group. More rats were randomised to the 5/6 nephrectomy group because the procedure was more extensive, and more rats were expected to die during surgery. The 5/6 nephrectomy procedure was carried out in two stages, initial (week -1) removal of approximately 2/3 of the left kidney together with insertion of a suprapubic catheter, followed one week later (week 0) by removal of the entire right kidney [2]. Sham rats also underwent a surgical procedure, however, no kidney tissue was removed. All surgical procedures were carried out under 2% isoflurane anaesthesia (Abbott Scandinavia, Solna, Sweden), and buprenorphine (Reckitt Benckiser, Slough, UK) was administered subcutaneously and via drinking water for pain relief. After surgery, rats had free access to tap water and standard rat chow (Altromin, Lage, Germany).

**Fig. 1:**
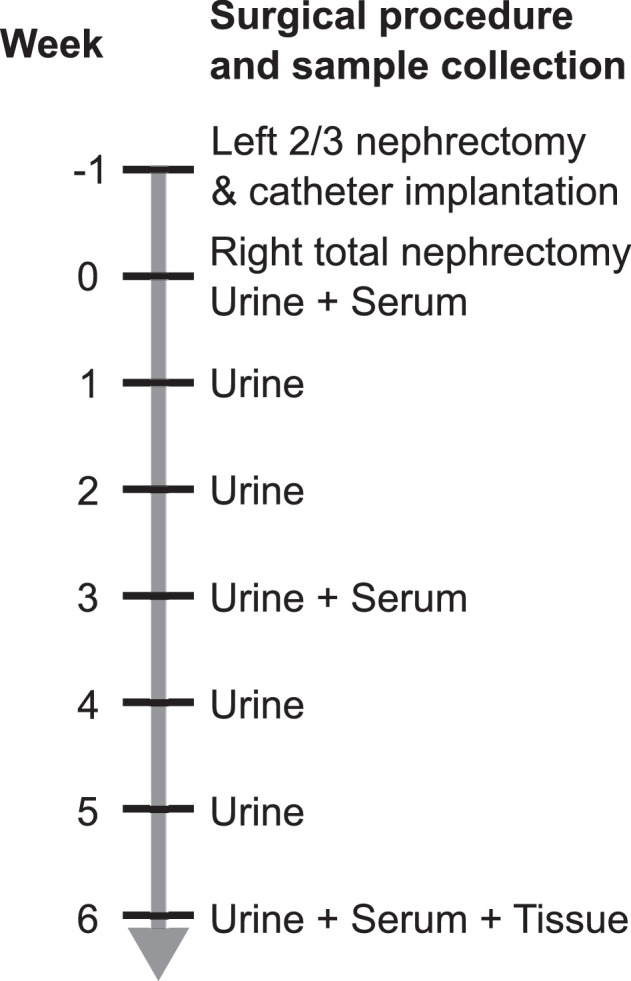
Sample collection protocol illustrating the timing of surgery, urine collection, serum collection, and tissue collection.

One week after the second operation and weekly thereafter (weeks 1–6 inclusive, see Fig. 1), a custom-built restraining cage was used to collect urine via a silicone tube connected from the suprapubic catheter into an Eppendorf tube suspended in a mixture of ice and water. During urine collection, rats were awake and had free access to water, whilst blood and tissue samples were collected whilst the rats were anesthetised. The tail vein was used to collect serum in weeks 0 and 3, although this proved difficult, and many serum samples are missing from these timepoints. Cardiac puncture was used to collect blood and obtain serum at the end of the experiment (week 6), and tissue was also collected at this point. Serum and urine were centrifuged and aliquoted, and all samples were frozen in liquid nitrogen and stored at -80°C until analysis.

### 2.2 Sample preparation and NMR analysis

Metabolomics protocols published by Beckonert et al. and Dona et al. were followed [3,4]. Urine and serum samples were left to thaw at 4 °C, vortexed briefly, and then centrifuged to remove precipitate (14000 x g and 4 °C for 5 min). Urine was mixed in a 9:1 ratio with buffer (1.5M KH_2_PO_4_, 2 mM NaN_3_ and 0.1% TSP dissolved in 99% D_2_O, pH* 7.4), whilst serum was mixed 1:1 with buffer (0.075M NaH_2_PO_4_, 0.04% NaN_3_ and 0.08% TSP dissolved in 20% D_2_O, pH* 7.4). Urine samples were re-centrifuged, because of precipitate formation after buffer addition, before transferring to an NMR tube for analysis.

Tissue samples were lyophilised and then homogenised with a Precellys homogenizer (Bertin Instruments, Montigny-le-Bretonneux, France). Metabolites were extracted using an ice-cold methanol, chloroform and water (2:2:1.8) protocol [5,6]. The hydrophilic phase was transferred to a clean vial, lyophilised, and then dissolved in 0.1 M imidazole-d_4_ buffer (0.02% NaN_3_ and 1 mM TSP dissolved in 99% D_2_O, pH* 7.0).

^1^H NMR spectra were recorded on a 600 MHz Bruker DRX-600 equipped with a TXI probe (Bruker BioSpin, Rheinstetten, Germany), using the following pulse programs: noesygppr1d, cpmgpr1d and jresgpprqf. Serum spectra were acquired at 310 K, whilst urine and tissue spectra were acquired at 298 K. Further acquisition details are listed in Table 2.

**Table 2:** Acquisition details for serum, urine and tissue spectra.

	1D NOESY	1D CPMG	J-resolved
Number of scans	128 64 (qNOESY)	128	4 (per increment)
Data points (time domain)	65536 (urine + tissue)	65536	Direct dimension: 16384 Indirect dimension: 42
Spectral width	20 ppm (urine + tissue)	20 ppm	Direct dimension: 12 ppm Indirect dimension: 54 Hz
Acquisition time	2.73 s	2.73 s	1.17 s
Receiver gain	90.5 (urine) 203 (tissue)	90.5 (serum + urine)	64 (serum + urine) 203 (tissue)
Relaxation delay	4 s 27.3 s (qNOESY)	4 s	4 s
B1 field strength	22 Hz (urine) 20 Hz (tissue)	17 Hz (serum) 22 Hz (urine) 20 Hz (tissue)	17 Hz (serum) 22 Hz (urine) 20 Hz (tissue)
Spectral size	131,072	131,072	Direct dimension: 32768 Indirect dimension: 64

### 2.3 Referencing and normalisation usage notes

Urine and tissue spectra have been referenced to the TSP signal, whilst serum spectra have been referenced to the α-H_1_-glucose duplet at 5.24 ppm, because TSP binds to blood proteins which can have an effect on the TSP integral and lineshape. No normalisation has been applied to the NMR spectra, however different normalisation strategies can be attempted: urine spectra can be normalised to the creatinine integral at approximately 3.05 ppm, or to total spectral intensity, due to widely varying urine concentrations; serum spectra can be normalised to the PULCON [7] signal at -2 ppm; and tissue spectra can be normalised to the TSP signal, followed by normalisation to extracted tissue mass (details of which are included in the MetaboLights sample table).

## Ethics Statement

Ethical approval for the animal experiments was provided by the Danish Ministry of Justice. The experiments were also conducted in accordance with the National Institute of Health “Guide for the Care and Use of Laboratory Animals” (8th edition).

## Contributions

Conceptualization and Resources: TR, SN, JF and RW. Methodology: TR, SN, JF, RW, MAH and MS. Investigation: MAH and MS. Software, Analysis, Data Curation and First Draft: MAH. Supervision: TR, RW, RGM and BSR. Review and Editing: All authors.

## Declaration of Competing Interest

The NMR laboratory at Aalborg University is supported by the Obel Family, SparNord and Carlsberg Foundations. Animal experiments were carried out at the Water and Salt Research Centre, Aarhus University, which was established and supported by the Danish National Research Foundation.
